# Analysing metaphorical pebbles in English RE

**DOI:** 10.1007/s40839-022-00181-x

**Published:** 2022-10-26

**Authors:** Paul Smalley

**Affiliations:** Faculty of Education, Edge Hill University, Ormskirk, UK

## Abstract

This paper is a critical analysis of the use of metaphors in English Religious Education (RE). It is grounded in the educational theory of Anna Sfard (a professor of Mathematics) who has written about learning metaphors, before considering some of the metaphoric language used in English education generally and RE specifically. It will examine carefully at how the metaphors implicit within the Commission on RE Report might help us interpret it, considering particularly the National Entitlement and note the changes to these metaphors in the newly adapted national entitlement in the REC Worldviews’ Project Draft Handbook. It will then turn (metaphorically) to the Research Review into RE from Ofsted and examine some aspects of that through metaphorical lenses. It will consider the metaphors inherent in Ofsted’s Deep Dive methodology, the work of Fordham, Counsell and the Early Career Framework and Core Content framework materials. The paper will conclude with some thoughts about RE as a subject discourse, suggesting that RE continues to have an identity crisis, which is not helped by a disciplinary, or a worldview, approach.

## Introduction

I had the privilege of watching an English Undergraduate trainee teacher recently who was teaching in a small school for secondary pupils with social & emotional problems, ADHD, autism, and associated conditions, who have endured serious difficulties at their previous schools. Three of the class were off on the day I visited, so the deputy Head and I, along with the class teacher watched my student teach one key stage 4 pupil, with the support of a Learning Support Assistant. The lesson was about using literary techniques to write creatively. It was an amazing lesson. The main part of the lesson began with some quick-fire quizzing about literary devices, one of which was a metaphor. The pupil, who was diagnosed as having an Autistic Spectrum Disorder, struggled at first to recall what a metaphor was, but then remembered, “it’s when you say something is something that it isn’t”.

This paper will carefully examine the metaphors habitually used in our subject, and in education more widely. It will consider some educational theory, using the work of Anna Sfard, before looking at the Commission on RE (CoRE) Final Report, *Religion and Worldviews*, (CoRE, 2018) and the implications of metaphors which have described its impact. It will examine carefully how the metaphors implicit within the Commission on RE Report might help us interpret it, considering particularly the National Entitlement, and briefly note some of the changes in metaphors in the latest iteration of that National Entitlement in Pett (2022). It will then turn (metaphorically) to the Research Review into RE from Ofsted (2021) and analyse some aspects of that through metaphorical lenses, considering Ofsted’s Deep Dive methodology and the Core Content Framework (CCF) and the Early Career Framework (ECF) before concluding with some thoughts about RE as a subject discourse.

## Sfard’s work on metaphors

Professor Anna Sfard is a mathematician at the University of Haifa and conducts research in the domain of learning sciences, with particular focus on the relation between thinking and communication. Incidentally she is Zygmunt Bauman’s daughter, the Polish born sociologist who conceived the concept of liquid modernity. I first came across Sfard’s work in a 1998 journal article entitled *On Two Metaphors for Learning and the Danger of Choosing Just One.* As one might expect, Sfard has a rather more sophisticated understanding of ‘metaphor’ than our earlier student.

In her chapter in *Educational theories, cultures and learning: A critical perspective* (Sfard, 2009) she suggests that metaphors are far more ubiquitously used in education than we might expect. She suggests that metaphors have four uses.

The primary way metaphors are used are as **discursive transplants**. I have a buddleia bush that originally grew at my sister’s in Wales, and we transplanted to our garden a few years ago – and to the other side of the garden earlier this year. The one plant has moved out of its original context. A metaphor does the same with language, moves it ‘out of its original context’.

Sfard goes on to suggest that far from being poetic embellishments metaphors are **catalysts of new knowledge.** Some of these expressions are so rooted in our everyday language we forget their metaphorical origins. Can we plant an idea? Does knowledge grow in the same way as the buddleia? She writes about *grasping an idea* – but no hands are used in that metaphorical grasping!

The learning paradox (first discussed by Plato) is this “how can we want to acquire knowledge of something which is not yet known to us? If we can only become cognizant of a thing by recognizing it on the basis of the knowledge we already possess, then nothing that does not yet belong to the assortment of the things we know can ever become one of them. Conclusion: creating new discourses – or knowledge – is inherently impossible.” (Sfard, 2009:40).

Sfard suggests that metaphors are the key to unlocking this paradox in that they use familiar concepts in an unfamiliar context to create a new understanding. This is similar to Gadamer’s (2004) discussion of prejudice as a precursor to understanding.

She goes so far to state that metaphors **Shape our thinking** - but does thinking really have a shape? Because we think that learning flowers in the young people we teach, that affects our concept of learning and education due to a whole range of horticultural metaphors. She quotes Plutarch ‘A mind is a fire to be kindled, not a vessel to be filled.’ And suggests the changing metaphor will affect our thinking – but more than that they **Shape our actions.** We will teach differently if we are kindling flames of filling vessels.

In their first year I introduce my undergraduates to the possibility that our understanding of education might be helped by considering the metaphor of a sponge. Often, they understand education as the process of instructing and imparting knowledge, which implies that the learner is passive. The sponge does not choose whether to soak up the water, it has no agency. Perhaps pupils are dry sponges, eager to draw forth water from any sort of curricular puddle or pool?

Maybe education is like this, involuntary soaking until examination time when all that knowledge is squeezed out of pupils in the summative assessment?

However, part of the process of educating involves the learner in making sense of the knowledge, which is imparted to him or her. Education is, therefore, a social activity and is premised on dialogue and relationship. It is not a transparent process but involves reflexivity. It follows that learning is the complex process of acquiring, constructing meaning from and assimilating knowledge. Key to this activity is recognising that learners bring with them experiences, values and that they belong to a social and cultural context, so the teacher’s role is to, “recognize what children already know in building plans for teaching and learning”, so they are able to, “build a window into the child’s mind” (Moon & Mayes, 1995: 23).

## Sfard’s two metaphors for learning

In 1998 Sfard published *On Two Metaphors for Learning and the Danger of Choosing Just One* in which she described learning as frequently divided into the acquisition metaphor and the participation metaphor. It is easy to misunderstand the two metaphors discussed by Sfard: participation does not simply mean groupwork, whilst acquisition means didactic teaching. Sfard’s article is much more about the nature of knowledge than the simple act of learning. It raises questions about whether learning is about becoming an active, knowledgeable participant in a community, which includes the teacher as an expert participant, with pupils learning to *be* something. Or are pupils recipients of the commodity of knowledge which the teacher has and is passed to, or shared with the pupil to individually enrich them?

If I think back to my time as a Football Coach - say coaching 5- and 6-year-olds, a viewer would see that the children were all engaged in playing football. So, in a simple game, where a series of footballs is moved from one area to another by dribbling the ball, whilst dodging other players, would look very much like participation metaphor. We were engaged in building up a team, and the players were being educated in how to be part of a football team - belonging, communicating and playing together, with the coaches as facilitators in a predominantly guided discovery mode of learning. However, maybe in reality I wanted the players to acquire the knowledge of how to do the technical skills of football, running, turning, dribbling.

Sfard (1998) suggests that for the most part, learning should be viewed as a combination of these two metaphors, participation and acquisition. When education is seen as simply one of these, and the current Hirschian vision permeating our educational philosophies is very much one which privileges the acquisition of knowledge as the only acceptable learning metaphor, then there are educational dangers inherent in that it may lead to the privileging of some groups to the disadvantage of others. Both metaphors, according to Sfard, have their advantages, and only by combining both, with their differing perspectives, can they liberate all who learn and teach.

## The metaphors of the Commission on RE

In 2018 the Religious Education Council of England and Wales (REC) published *Religion and Worldviews*, a report produced by fourteen independent expert professionals, which made a number of recommendations for the future of RE in England (see Tharani in Chater 2020 for further detail). Trevor Cooling was the Chair of the REC throughout the process and has repeatedly stated that the CoRE report should be viewed as a pebble in the pond to cause ripples and not commandments written on tablets of stone – and he has also claimed that he hoped that it will be a gamechanger (Salter, 2021). Each of these three notions are of course metaphors and will be considered in turn.

The CoRE report is not a tablet of stone. This is of course a biblical metaphor. The intention of those who use it, is that it brings up ideas of authority (the tablets being given to Moses by God), permanence and uniqueness – which handily forgets that the original tablets were broken and the ones carried around the Sinai were version two (Exodus 31–34). Cooling, by rejecting this metaphor seems to suggest that the Commission report is not divinely inspired, unchangeable, uniquely authoritative or permanent.

The CoRE report is a pebble thrown in a pond. If one imagines the act of throwing pebbles in a pond, those pebbles are relatively lightweight, and usually found lying around the pond. Whilst a pebble thrown may cause some temporary disruption to the pond, sending out ripples, the pond quickly returns to its natural still state. Perhaps Cooling is suggesting that there are many pebbles which have been (and probably will be) thrown in the RE pond? In the last decade, the REC (2013) *Curriculum Framework*, the Commission on Religion and Belief in Public Life (CoRAB, 2015), RE for Real (Dinham & Shaw, 2015), A New Settlement (Clarke & Woodhead, 2015, 2018), Mark Chater’s Reforming RE (Chater, 2020), all could be considered as pebbles. But no matter how many pebbles are thrown, there is no lasting change to the pond itself. Cooling hasn’t written using the pebble metaphor, but in an email he stated:

*I use it [the pebble metaphor] because soon after CoRE was published, I realised that people were seeing CoRE as like government guidance rather than a stimulus to an educationally game-changing conversation. In my view that would kill it because there were clearly elements … that needed further development. My use of the pebble analogy did cause some agitation as others thought we should treat the CoRE Report like Moses’ tablets of stone.* (Cooling, 2021)

Cooling had used the game-changer metaphor from the very start of the Commission process:

*On several occasions when asked about CoRE I have made reference to Schools Council Working Paper 36 published in 1971. This is the closest parallel to my aspiration for CoRE that I can think of. It was a ****game-changer ****in its impact and framed the modern approach to RE with its championing of a then pioneering multifaith approach. This had huge consequences for both teachers and policymakers. Forty-five years is a long time to wait for another report of such significant impact. My aspiration is that CoRE will fulfil that role.* (Cooling 2016)

“Game-changer” can mean one of two things: a person or occurrence that changes the rules, the substance, the nature of the game (such as the mythical picking up of the ball by William Webb Ellis, who changed the game of football into rugby) or a person or occurrence that changes the outcome of a game (such as any number of heroic saves by a goalkeeper at a crucial time in a football match). It is therefore appropriate to consider what sort of metaphorical gamechanger was Cooling hoping the CoRE would be. Smart’s Working Paper can rightly be considered a Webb-Ellis game-changer. In many ways it led to a sort of rugby-soccer split in English RE: with (at least some) schools (often) with a religious character playing RE by the old theological/biblical rules whilst most other schools adopted (often quite a poorly constructed) world religions/phenomenological RE. Perhaps the RE community’s perception of CoRE was clouded by their perception that Smart’s impact was to move from RE being about one religion - to being about more religions. Because many forget that Working Paper 36 (Schools Council, 1971) was a change from a theological paradigm to a phenomenological paradigm they have been blind to seeing CoRE as a ‘paradigm shift’ from a weakly world religions approach to a Worldview approach, not an inclusion of additional (non-religious) beliefs. When people talk of a worldview approach they are using that term metaphorically, discursively transplanting a term in order to change our thinking and our actions.

On the one hand, as Nietzsche argued (Hinman, 1982), all language is metaphorical: it is representative of something which it isn’t. In fact, Nietzsche argued that metaphoric meaning takes precedence over literal meaning. Language is socially constructed, fluid and only makes sense to people who are in agreement with those who have constructed that meaning. When my children were small – at night they would lie in their bedrooms and look at their mobiles. Now they are older they lie on their beds and look at their mobiles. The words have not changed but the meaning is fluid. This is certainly thought to be the case with the name of the subject. I’ve written before that the perpetual discussion of the name of the subject does nothing to enhance the perception of the subject (Smalley, 2020). Instead, I would much rather we embrace something of the new vision of RE from the CoRE Report and evolve new meaning into the name of RE.

## The metaphor of CoRE’s new vision for RE

John Hall in the Foreword to the CoRE report suggests in a rather vague statement that, “We offer a new vision: the subject should explore the role that religious and non-religious worldviews play in all human life.” (CoRE, 2018: i) Appendix 1 states: “It is about understanding the human quest for meaning, being prepared for life in a diverse world and having space to reflect on one’s own worldview” (CoRE, 2018: 73). There is something to ponder on Pat Hannam and Gert Biesta’s (2019) critique that the Worldview paradigm is a philosophical metaphor – a hermeneutic metaphor – that objectifies understanding and one which does not fully appreciate the existential nature of religious belonging.

Notwithstanding that critique, this vision of RE as a quest for human understanding is what the best RE curriculums and the best RE teachers have been doing for years. For example, the central question of the Lancashire Agreed Syllabus from at least the 2011 syllabus – it is “what does it mean to be human?” This is not a propositional statement, but an existential question.

This is a vision I think I can get behind: (noting the metaphorical complexity of being positioned behind a vision) pupils should study the ways secular and sacred worldviews have used narrative, questions, symbols and praxis (Hella, 2009) to try to make sense of the world, both through history and in contemporary society. I do wonder if that four-fold taxonomy of knowledges that are fundamental to the study of a worldview sufficiently takes account of Pat Hannam’s (2018) conceptualisation of religion as propositional belief, practice and the existential.

If pupils understand the ways that these secular and sacred worldviews relate and inform the fluid worldviews of individuals in society, causing people to believe or behave in certain ways, it will prepare them for the contemporary liquid modernity (Bauman, 2000) which they inhabit. And through all this learning, if given space for personal reflection, pupils will have opportunity to engage in epistemic cognition (Fetz & Reich, 1989) and develop their own emerging personal existential place in the world. This is the sort of RE that I have encouraged those beginning RE teachers who have trained with us at Edge Hill to explore.

The central plank of the CoRE is in many ways the proposed National Entitlement statement (CoRE, 2018: 12–13). This entitlement statement is largely metaphor free, but it leads more easily to a conception of learning as acquisition than learning as participation. It states things: discrete (or not so discrete) objectified elements of knowledge that are collected together and ‘must be taught’. It suggests we teach “about key concepts such as ‘religion’…” not that we teach “what it means to be religious”. Hannam’s critique of the 2013 RE Council Framework is apposite:

*My point is that not only has this led to an overemphasis on learning in religious education, but also to an objectification of religious knowledge with accompanying risks of knowledge so understood becoming separated from what it actually means for someone to live a religious life.* (Hannam, 2018: 29)

Note too, that the teacher is the actor in the statement – pupils don’t need to understand – or even learn – anything, they simply must be taught. The entitlement statement in the CoRE report reads as a series of large, objectified lumps of curricular ‘stuff’ that we wise teachers possess and deliver to our willing pupils.

The latest phase of the REC’s worldviews project is a “the creation of a toolkit of materials to help subject leads and advisers build a syllabus and define a curriculum for teaching religious education framed by the new worldview vision.” (RE Today, 2021) This work is being done in three phases – the first being the creation of a draft handbook, or curriculum resource authored by Stephen Pett, to inform teams of syllabus writers who will produce exemplar frameworks (phase 2) and resources (phase 3). The Draft Curriculum Resource (Pett, 2022) includes a revision of the national statement of entitlement (NSE). I am pleased that Pett appears to have taken on board my observations about the language of the 2018 version. The revised NSE states that ‘pupils must be taught to understand’ (Pett, 2022: 19) not simply ‘taught’ (CoRE, 2018, 12). Pett deliberately uses a metaphor of a mixing desk to try to explain how the various curricular elements of the NSE might be blended in differing units of work. (2022, 37). However, there is not space in this paper for a full analysis of this draft document, which will be re-drafted and republished in 2024.

## Ofsted’s use of metaphors

Perhaps the next pebble, or tablet of stone, is Ofsted’s RE Research Review. Ofsted are the school inspection service in England and have a primary responsibility for ensuring schools (and other services) are functioning well. They have recently set up a ‘curriculum division’ appointing subject specialist advisers who have been asked to survey existing literature and publish reports that define what ‘high quality provision’ might feature in each subject. The Research Review into RE was one of the first to be published in May 2021. We could debate what the best petrographic metaphor for this publication is. I would suggest that it is more than a pebble, perhaps a boulder, since I would suggest that it will be more influential that the Commission Report in shaping our school RE curriculums. Within it, there are some key metaphors which require examination.

One thing that is clear from the report, and incidentally the other Research reports that Ofsted have published, is that Ofsted see the curriculum as a journey. This is of course another metaphor, and it is right to consider what sort of journey Ofsted is thinking of when it uses this metaphor.

Ofsted do not see the curriculum as a cruise, where, sitting on a luxurious boat holidaymakers are wined and dined, stopping off at various Mediterranean ports to see the sights of Barcelona, Monte Carlo, Rome or wherever. Perhaps this is the sort of journey that metaphorically aligns to the world religions paradigm: the Cook’s tour of world religions to repeat the criticism of Smart’s phenomenological approach quoted by Bastide (1992), Gearon (2013) and others?

Ofsted is imagining the sort of journey I took one weekend when I went to London. We started with an end point (not particularly ambitious, but an end point, a West End theatre show), we considered various methods of getting there (train or car) and decided car was the better option. We planned a route (down the M6). We responded to formative assessment along the way (Google maps told us that there was congestion ahead and we took the toll road) and we arrived at our destination hotel. However, this was only the end of a key stage of the journey, as we then took the tube to get into Central London and see the show.

Another metaphorical statement is that “The curriculum is the progression model.” (Ofsted, 2019: 7). Educators have become so used to this sort of language that they may forget that ‘model’ is metaphorical. Fordham (2020) claims to have invented the phrase (with Christine Counsell) in around 2014. It grew out of a dissatisfaction with level based assessments and those that were replicating it, or GCSE questioning as a way of measuring progress. And he states: “*The problem, rather, was that mark schemes do not describe the journey one needs to go on to get from one level to the next.”* He, drawing on Daisy Christodoulou’s (2016) oft repeated analogy of a marathon runner not getting better at marathons by simply doing more marathons, suggests that:

*This is why mark schemes (i.e. accounts of summative performance) cannot tell us as teachers what to do to help a pupil get better at our subject. A well-designed summative assessment might be able to tell us with some degree of accuracy whether or not a pupil has got better at something, but that assessment cannot inform how to get better.*

One of the big ripples from the Research Reviews published recently may be a much stronger distinction between formative and summative assessment. For too many years, I think, the demands on teachers to use formative assessment as a progress measure, and to use summative assessments formatively has led to confusion about the nature and purpose of assessments.

But we are being distracted: Fordham states that the model is a journey – our two metaphors are the same. He bows to Counsell (2018: np) to explain what sort of journey the model is. She reminds us that Curriculum is itself metaphorical, originally deriving “from the Latin ‘currere’ meaning a race or a course on which a race is run. The Latin verb ‘currere’ means to ‘run’ or ‘proceed’. The word is replete with a sense of movement.” She goes on to suggest this is helpful as curriculum is a journey of a specified distance, structured over time, like a novel, film symphony or some other narrative form. She doesn’t explore the idea that a race is predicating on one person winning and everyone else losing.

So somewhat mischievously “the curriculum is the progression model” could be reworded as “the journey is the journey journey”. However, there is value in the curriculum- as- narrative metaphor: the idea that multiple plot lines and characters develop over time to make a cohesive ‘sense’ is appealing to me. It would be perverse to argue with an intention that RE pupils will know, remember and be able to do more as they do their RE lessons.

All of the research reviews published so far have a section defining types of knowledge in those subject areas, and they are similar – but different:

Science has substantive knowledge and disciplinary knowledge.

Maths has declarative, procedural and conditional knowledge – which are simply categories of the substantive.

Languages interestingly calls them pillars: phonics, vocabulary and grammar.

Geography has a diagram, dividing substantive knowledge into four distinct parts and showing disciplinary knowledge as overlapping them.

RE is the only one with a separate category of personal knowledge, “where pupils build an awareness of their own presuppositions and values about the religious and non-religious traditions they study” (Ofsted, 2021: np). This ‘personal knowledge’ appears very similar to the idea of Attainment Target 2, Learning from religions prevalent in RE curricula of the 1990s and early 2000s (Teece, 2010). It also seems similar to that CoRE aim of “having space to reflect on one’s own worldview” (CoRE, 2018: 73).

David Lewin and Janet Orchard (2021) have rightly questioned why this is a category of knowledge when it could be viewed simply as understanding substantive and disciplinary knowledge. They too raise the issue that Hannam was concerned with, that RE becomes only concerned with the cognitive dimension of human experience. One further critique of theirs which I wish to highlight is that despite the Research Review stating that “In high-quality RE curriculums, these three types of knowledge are not artificially separated from each other”, there is a tendency in the report to treat them as such, with pre-existing substantive content being chosen and the tools of a disciplinary approach being chosen to analyse it. Perhaps an overlapping diagram like geography might help? But there is a wider hermeneutical question of how the objects of substantive knowledge have been formed by the disciplines which have formed them.

Ofsted must have been thinking of the RE curriculum as a pond, as the Research Report requires representations to be both deep and broad. Given that the categories of “religion and non-religion” collective encompass the totality of the known universe and beyond, the potential size of the substantive knowledge required “for pupils to build up accurate knowledge of the complexity and diversity of global religion and non-religion.” (Ofsted, 2021: np) is infinite. Despite the fact that legislation in England “requires the [RE] syllabus to reflect that the religious traditions of Great Britain are in the main Christian whilst taking account of the teaching and practices of the other principal religions represented in Great Britain.” (Education Act, 1996), the report hints that Ofsted will consider a curriculum sufficiently broad, even if not all of the ‘big six’ feature in a school’s curriculum (“For example, if depth of study takes place only in Abrahamic traditions (Jewish, Christian and Islamic) and no dharmic traditions, then pupils’ schema of ‘religion’ would be skewed.”), but leaves it to leaders at the school level to make these decisions. There is no specificity of which elements of the possible substantive knowledge should be studied to make up sufficiently enough depth and breadth in the RE pond.

There is some specificity in the suggested ways of knowing:

*“Of course, a school RE curriculum could never fully capture every method, tool or process that could be used concerning religion.”* (Ofsted 2021: np)

A range of tools are suggested, textual analysis, ethnography, data analysis, interviews and so on, but schools are expected to know why these tools are being used, as well as developing the knowledge to use them in RE. In the Ofsted Research Report into RE, one simple way of structuring these academic conversations is mentioned:

*“Some curriculum approaches formalise ‘ways of knowing’ into simplified disciplines, such as ‘theology’, ‘philosophy’ and ‘human/social sciences’.”* (Ofsted 2021: np)

In England RE curricula are decided on a local basis and there are therefore over 150 ‘Agreed Syllabuses for RE’ (although many of them are shared). At the time of the publication of the Ofsted Research Review, this threefold division of ways of knowing aligned perfectly with the way only one of those Agreed Syllabuses was structured – and one the co-incidentally that the author of the Ofsted Report was involved in producing (Norfolk, 2019). It is true that the report states that high quality RE *may* have a curriculum design that includes ‘ways of knowing’ and is only in the report as a suggestion, an exemplar, but I think it places schools in 150 authorities with a problem, if their current curriculum, based on a local agreed syllabus does not teach the ‘ways of knowledge’ intrinsic to this recommendation. In the time since publication, this has led to the rapid inclusion of the ‘ways of knowing’ into syllabus revisions (e.g., Lancashire, 2021).

## Ofsted’s deep dive metaphor

Since the 2019 Ofsted inspection framework was published, Ofsted’s method of conducting inspections has been to negotiate with the school a range of subjects for the inspection to focus upon. Ofsted have been clear that these are not inspections of the subject deeply dived into, but as exemplars of the systemic features of the school curriculum. The idea is that if an inspector immerses herself in a particular subject area for a day and discovers that it is planned, taught and assessed well, this would indicate that this is likely to be true for all curriculum subjects in the school, and also indicate that the school as a whole is well managed. Others might claim that the school might encourage the inspector to deep dive into ‘strong subjects’ and stay away from the more problematic curriculum areas.

An examination of the first 234 Ofsted Reports on secondary schools published between October 2019 (the start of the new framework which included the deep dive methodology) and February 2020 (when inspections were disrupted by the COVID-19 pandemic) shows that only 3% (seven schools of the 234) of inspections under the new framework deep dived into RE, while the overwhelming majority of secondary schools had inspectors deep dive into English (222, 95%), mathematics (187, 80%) and the other EBacc subjects (Science 164, 70%, Languages 119, 51%, History 113, 48%, Geography 84, 36%). Ofsted grades schools according to four grades: Outstanding, Good, Requires Improvement, Inadequate. In the sample, 63% were awarded the top two grades. In the seven schools where RE was focussed upon, this dropped to 43%. This might suggest that the methodology is working and where there are problems with RE curriculum, this is evidence of the ‘systemic failure’ to provide a broad, balanced and ambitious curriculum, not a failure to provide a decent RE curriculum.

## Core Content Framework / Early Career Framework

My last pebble, and one that I am metaphorically skimming over the surface of the pond, is the Core Content Framework (CCF) and/or the Early Career Framework (ECF) (Department for Education, 2019). “The early career framework sets out what early career teachers are entitled to learn about and learn how to do when they start their careers” (Department for Education 2019: np). The two documents list an identical series of propositional statements that teachers (or in the case of the Core Content Framework, trainee teachers) must learn that and learn how to….

For example, on subject and curriculum, there is nothing subject specific, but teachers must learn that point “2. Secure subject knowledge helps teachers to motivate pupils and teach effectively” and they should learn how to “Deliver a carefully sequenced and coherent curriculum, by:” doing the things listed. It is based on a wealth of research material that is in line with much of the Gibb/Gove/Hirsch thrust of recent government education policy; for example, it accepts cognitive science theories as gospel, and advocates adaptive teaching over differentiation. It states that teachers must learn how to interleave “concrete and abstract examples, slowly withdrawing concrete examples and drawing attention to the underlying structure of problems.”

The Ofsted RE Research Review has trodden a careful line on such matters. Whilst the research review states there are a variety of ways of delivering a high quality RE curriculum, the CCF/ECF states that there is one way that teachers must learn. For example, in the CCF/ECF, teachers *must* (emphasis mine) learn how to develop fluency, by using quizzing and retrieval practice to build automatic recall of key knowledge, whereas in the RE research review we are told that low stakes, multiple choice quizzes might be useful for checking vocabulary, but not for recalling narratives.

Careful reading of the CCF/ECF materials indicates much can be aligned to what Sfard would call the acquisition metaphor of learning, and very little participation. The very nature of a list of mandated propositional statements is a clear indication of that understanding of knowledge that is known by experts and acquired by novices.

## Conclusion

This paper has thought quite deeply about metaphors in education and considered how a number of them that relate to the RE curricular pond. It considered whether the CoRE report was a Tablet of Stone or a pebble – and the implications of those metaphors. It wondered about what sort of game-changer Smart’s Working Paper and the CoRE report might be. Considered the name of the subject and the slippery nature of language. It has looked carefully at the metaphors implicit within the CoRE Report and considered whether both the CoRE National Entitlement and Pett’s revised Entitlement understood learning as acquisition, participation or both.

It turned to the boulder of the Research Review from Ofsted and examined some aspects of that through our metaphorical lens, considering journeys and races and narratives, and the different ways knowledge has been categorised. It thought about the metaphor of Ofsted’s Deep Dive methodology and briefly thought how the skimming stone of the ECF/CCF might effect classroom practice. It now concludes by asking somewhat rhetorical questions and considering if Sfard’s understanding of subject discourse might provide a solution to the identity crisis of the subject which the CoRE report, the Worldviews project and the Ofsted Research Review have all missed.

Sfard (2020), who incidentally is not referenced in any of the Research Reviews, conceptualises subjects as special forms of communication, or discourses. Taking her specialism, mathematics, as the example she suggests that each subject discourse has its own particular set of keywords, visual mediators, routines and ‘generally endorsed narratives’. She states that:

*“thinking mathematically (scientifically, sociologically) means participating in the historically developed discourse known as mathematical (scientific, sociological).”* (Sfard, 2020: 90)

The Geography Research Review has a section on ‘Thinking like a geographer’.

It draws on published geography research to show this means how pupils can learn to:

- use what they know from one context in another;
- think about alternative futures;
- consider their influence on decisions that will be made.

Our subject once again finds itself with an identity crisis. What particular set of keywords, visual mediators, routines and ‘generally endorsed narratives’ does RE have that sets it apart from other discourses? The Ofsted Research review dodges this by offering other discourses - theology, philosophy and social sciences - as academic conversations; but is that really what the subject is – just a combination of other discourses? What would it mean to think like a religious educationer? We don’t even have the language! Unless we are willing to make a grand claim that RE is about the quest for meaning which is implicit in the state of being human. Perhaps being good at our subject means participating in the historically developed discourse known as being human. Now that’s an ambitious end goal for a curriculum!

## References

[CR1] Bastide, D. (1992). *Good practice in primary religious education 4–11* (p. 98). Falmer Press

[CR2] Bauman Z (2000). Liquid modernity.

[CR3] Chater, M. (2020). *Reforming RE: Power and Knowledge in a Worldviews Curriculum*. John Catt: Woodbridge

[CR4] Christodoulou, D. (2016). *Making good progress?: the future of assessment for learning*. Oxford University Press

[CR5] Clarke C, Woodhead L (2015). A New Settlement: Religion and belief in schools.

[CR6] Clarke C, Woodhead L (2018). A New Settlement Revised: Religion and belief in schools.

[CR7] Commission on Religious Education (CoRE), (2018). *Religion and Worldviews: The Way Forward*. Religious Education Council of England and Wales

[CR8] Cooling, T. (2021). private email correspondence

[CR9] Cooling, T. (2016). Commission on Religious Education, REC https://www.religiouseducationcouncil.org.uk/news/commission-on-religious-education/

[CR10] CORAB (2015). *Living with Difference: Community, Diversity and the Common Good* https://corablivingwithdifference.files.wordpress.com/2015/12/living-with-difference-online.pdf

[CR11] Counsell, C. (2018). *Senior Curriculum Leadership 1: The indirect manifestation of knowledge: (A) curriculum as narrative*, https://thedignityofthethingblog.wordpress.com/2018/04/07/senior-curriculum-leadership-1-the-indirect-manifestation-of-knowledge-a-curriculum-as-narrative/

[CR12] Department for Education (2019). *ITT Core Content Framework*. https://www.gov.uk/government/publications/initial-teacher-training-itt-core-content-framework

[CR13] Department for Education (2019). *The Early Career Framework*. https://www.gov.uk/government/publications/early-career-framework

[CR14] Dinham, A., & Shaw, M. (2015). *RE for REal: The future of teaching and learning about Religion and belief* https://www.gold.ac.uk/media/goldsmiths/169-images/departments/research-units/faiths-unit/REforREal-web-b.pdf

[CR15] Fetz RL, Reich KH (1989). World Views and Religions Development. Journal of Empirical Theology.

[CR16] Fordham, M. (2020). *What did I mean by ‘the curriculum is the progression model’?* https://clioetcetera.com/2020/02/08/what-did-i-mean-by-the-curriculum-is-the-progression-model/

[CR17] Gadamer HG (2004). Truth and method.

[CR18] Gearon L (2013). MasterClass in religious education: Transforming teaching and learning.

[CR19] British Government (1996). *Education Act*, c 56

[CR20] Hannam, P. (2018). *Religious education and the public sphere* (1st ed., Ser. Theorizing education). Routledge

[CR21] Hella E (2009). Developing Students’ Worldview Literacy through Variation: Pedagogical Prospects of Critical Religious Education and the Variation Theory of Learning for Further Education. Journal of Chaplaincy in Further Education.

[CR22] Hinman, L. M. (1982). Nietzsche, Metaphor, and Truth *Philosophy and Phenomenological Research* Vol. XLIII, No. 2, 179–199

[CR23] Lancashire (2021). *Lancashire Agreed Syllabus for Religious Education Searching for Meaning What is it to be human?* Lancashire

[CR24] Lewin, D., & Orchard, J. (2021). *What’s ‘what’ in RE: Relating the what, the how and the why of curriculum content*. https://butterfly-butterfly-bnhl.squarespace.com/blog/whats-what-in-re-relating-the-what-the-how-and-the-why-of-curriculum-content?fbclid=IwAR2iTMCXnmphP9hNuxFO2DRZhbW9IB5tE6JJ4pXo4dVhpBGKaVJEAQ4u2fo

[CR25] Moon B, Mayes AS (1995). Teaching and Learning in the Secondary School.

[CR26] Norfolk (2019). *A Religious Education for the Future: Understanding religion and worldviews for a life in a changing world* https://www.schools.norfolk.gov.uk/-/media/schools/files/teaching-and-learning/religious-education-agreed-syllabus/norfolk-religious-education-agreed-syllabus-2019.pdf

[CR27] Ofsted (2019). *Education inspection framework (EIF)* https://www.gov.uk/government/publications/education-inspection-framework/education-inspection-framework

[CR28] Ofsted (2021). *Research review series: religious education* https://www.gov.uk/government/publications/research-review-series-religious-education

[CR29] Hannam P, Biesta G (2019). Religious education, a matter of understanding? reflections on the final report of the Commission on Religious Education. Journal of Beliefs & Values.

[CR30] Pett, S. (2022). *Religion and Worldviews in the Classroom: developing a Worldviews Approach* https://www.religiouseducationcouncil.org.uk/wp-content/uploads/2022/07/REC-Worldviews-Project-double-pages-Revised.pdf

[CR31] RE Today (2021). Press release, available from https://www.retoday.org.uk/news/latest-news/templeton-grant-award-project/

[CR32] Religious Education Council of England and Wales (REC). (2013). *A Curriculum Framework for Religious Education in England*. London: RE Council of England and Wales. http://www.reonline.org.uk/wordpress/wp-content/uploads/2015/03/RE_Review_Summary-CurriculumFramework.pdf

[CR33] Salter E (2021). A critical reflection on the Commission on Religious Education’s proposed National Entitlement to Religion and Worldviews in England and Wales. Journal of Religious Education.

[CR34] Schools Council (1971). *Working Paper 36: Religious Education in Secondary Schools*. London: Evans/Methuen

[CR35] Sfard, A. (2009). Metaphors in education. In H. Daniels, J. Porter, & H. Lauder (Eds.), *Educational theories, cultures and learning: A critical perspective* (pp. 39–49). London Routledge

[CR36] Sfard A, Mercer N, Wegerif R, Major L (2020). Learning, discursive fault lines, and dialogic engagement. The Routledge International Handbook of Research on Dialogic Education.

[CR37] Sfard, A. (1998). On Two Metaphors for Learning and the Danger of Choosing Just One. *Educational researcher*, *27*(4), DOI: 10.2307/1176193

[CR38] Smalley, P. (2020). “Reflections on *The Way Forward*: A neoliberal future for RE in England?” *Professional REflection: Theory and practice. 37, 3*

[CR39] Teece G (2010). Is it learning about and from religions, religion or religious education? And is it any wonder some teachers don’t get it?. British Journal of Religious Education.

